# Strawberry varieties differ in pollinator‐relevant floral traits

**DOI:** 10.1002/ece3.10914

**Published:** 2024-02-05

**Authors:** Hamish A. Symington, Beverley J. Glover

**Affiliations:** ^1^ Department of Plant Sciences University of Cambridge Cambridge UK

**Keywords:** bumblebees, floral reward, nectar, pollen, pollination, strawberry

## Abstract

A rising global population will need more food, increasing demand for insect pollination services. However, general insect declines conflict with this demand. One way to mitigate this conflict is to grow crop flowers that are easier for insects to find and more rewarding to those that visit them. This study quantifies variation in the pollinator‐relevant traits of nectar and pollen production, flower size and flower shape in commercial strawberry, finding significant variation between varieties in all traits. Bumblebees could learn to distinguish between the extremes of variation in flower shape, but this learning is very slow, indicating that this variation is at the limit of that which can be detected by bumblebees. Bee preferences for nectar of differing sugar concentrations at field‐realistic volumes were consistent with previous observations at larger volumes, suggesting that it is valid to translate lab findings to the field. This study builds on our knowledge of the range of pollinator reward present in a single cultivated species and of the impact of field‐realistic levels of variation in floral traits on bumblebee preferences.

## Graphical abstract

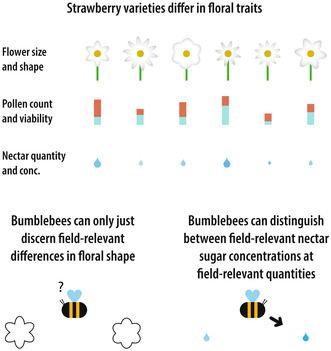

## 1 INTRODUCTION

Pollinating insects are a key component of modern agriculture, implicated in the production of around 75% of the 115 most important crops (Klein et al., 2007). Even though many other crops set at least some fruit when insects are excluded, insect pollination is essential for some crops and improves yield or fruit/seed quality—and, therefore, economic value—in many species (Klatt et al., 2014; Klein et al., 2007; Lieten, 1994).

Various factors are contributing to a ‘perfect storm’ of issues surrounding pollinator‐dependent crop production. The pollinator dependency of agriculture is rising (Aizen & Harder, 2009) and an increasing global population (population.un.org, 2022) will require more food to be produced. Even though numbers of honeybee colonies are rising (FAO, 2022), populations of many wild pollinator species are declining (Kluser & Peduzzi, 2007; Potts et al., 2016) for numerous reasons (Dicks et al., 2021). This is significant because pollination by wild insects improves crop productivity over and above pollination by honeybees (Garibaldi et al., 2013; Greenleaf & Kremen, 2006; MacInnis & Forrest, 2019). This has led to concerns about a ‘pollinator crisis’ threatening food supply (Potts et al., 2010; Steffan‐Dewenter et al., 2005). One mitigating strategy which has received little attention is to focus on the plants. Plant‐based approaches include lowering the plant's overall demand for pollinators by increasing parthenocarpy or autogamy, or by switching from animal to wind pollination; making flowers easier for insects to find or handle, thus increasing the number of flowers which can be pollinated per unit time; altering floral architecture to make pollen transfer more efficient or more likely; or altering the reward which the flowers offer, to provide more energy or nutrition for the insects, to optimise the energetic reward for collection efficiency (Pattrick et al., 2020), or to include bioactive compounds which enhance bee performance (Wright et al., 2013). Improving the energy, nutrition or collection efficiency of the reward would result in better provisioning of colonies, enhancing pollinator populations in the longer term as well as crop yield in the short term. The strategies of making flowers easier to find and improving reward are not mutually exclusive: it would be possible to select for traits contributing to both strategies, offering the exciting potential for combinatorial improvement.

Pollinators are attracted to flowers by an interplay of numerous stimuli, both visual and olfactory (Chittka & Raine, 2006). Studies using bumblebees as a model organism representative of pollinators have shown that they generally prefer larger flowers (Galen & Newport, 1987; Stanton & Preston, 1988), but this may be because larger flowers are easier to see, but also perhaps that larger flowers may produce more by way of a nectar reward (e.g. Cresswell & Galen, 1991; Eisen et al., 2023; Stanton & Preston, 1988). It has been shown in strawberry that pollinators prefer longer petals (Ashman, 2000), and there are numerous experiments investigating bee responses to shape and pattern (Lehrer et al. (1995) and see parts of Hempel de Ibarra et al. (2014) for a review of honeybee vision).

Once the pollinator is on the flower, numerous other traits can affect reward access and pollination efficiency, including but not limited to corolla width (particularly in tubular flowers), positioning of stigma and anthers, or the presence of nectar spurs, which can be so restrictive to pollinators as to drive evolutionary change (Whittall & Hodges, 2007). From the pollinator's point of view, the key considerations are the availability of nectar and/or pollen rewards presented by the plant, in terms of quantity, quality and accessibility.

Nectar is a mix of several different sugars, amino acids, alkaloids, proteins, minerals and more (Nicolson et al., 2007), and its composition (both in terms of quantity of sugar and presence or absence of other components) affects pollinator preference (Baracchi et al., 2017; Wykes, 1952). Numerous studies have tested bee nectar preference: examples include nectar concentration (Bailes et al., 2018), concentration and volume together (Cnaani et al., 2006) and sugar composition (Wykes, 1952). From these studies, it is clear that, if sugar compounds are standardised, bee preferences are driven more by nectar sugar concentration than by volume.

Pollen is the primary protein source for honeybees and bumblebees. There are relatively few studies investigating bumblebee pollen preferences (see Section 4). One of these showed that the number of viable pollen grains is the single most important predictor of bumblebee behaviour (Robertson et al., 1999). Having a larger percentage of viable pollen will also improve autonomous self‐pollination or animal‐mediated pollination in a flower. Both honeybees and bumblebees select the species of plant from which they wish to collect pollen, rather than collecting indiscriminately, with bumblebees being more discerning, focusing on quality rather than quantity (Leonhardt & Blüthgen, 2012) and selecting plants based on the ratio of protein to lipid in the pollen (Vaudo et al., 2016).

Strawberry (*Fragaria* × *ananassa*) is a crop whose yield and quality are improved by insect pollination (Free, 1993). Production is commercially of significance, with 2020 worldwide harvests of 8.9 m tonnes, accounting for 72% of the berry production worldwide (81% in the UK, with raspberries, the next most abundant crop, accounting for 8%; FAO, 2022). As for many other crops, commercial breeding has selected for numerous producer‐ and consumer‐relevant traits, including cropping time, growth conditions, disease and pest resistance, size, taste and shelf life and hundreds of varieties are commercially available.

This study uses the commercial strawberry as a model organism to explore the potential for improving flowers to benefit pollinators. The aims are to identify whether variation exists between varieties in pollinator‐relevant floral traits (flower size and shape, nectar and pollen reward) and, if so, whether those traits are discernible by and/or useful to bumblebees. Given the wide range of potential targets for improvement we focus on flower shape, nectar quantity and sugar concentration and pollen count and percentage viability: these traits are the most practical to measure across a relatively large number of varieties and to explore using bumblebee behavioural experiments. Production of volatile compounds has previously been studied in some varieties of strawberry (Ceuppens et al., 2015; Mozūraitis et al., 2020) and does not form part of this study.

The data we report build on our knowledge of the range of pollinator reward present in a single species and of the impact of field‐realistic levels of variation in floral traits on bumblebee preferences.

## 2 METHODS

### 2.1 Study species and growth conditions

*F*. × *ananassa* plants were supplied by commercial strawberry farmers RW Walpole (Terrington St Clement, UK). They were June‐bearing varieties grown by or available from them during the study, and all were from virus‐free stock. For Field Study 1, plants of 21 varieties were grown by RW Walpole in a commercial plant production field (see Figure A1, Table A1 and the Appendix S1 for variety list, field specification and herbicide application).

It was not possible to collect nectar and pollen samples during Field Study 1 due to the short time period during which the plants were flowering, the presence of fine soil grit impeding nectar collection, and the impracticality of maintaining 21 separate pollinator exclusion cages in a commercial field in which tractors were used for cultivation. Nectar and pollen quantification was, therefore, undertaken in a separate field study in a dedicated polytunnel. For Field Study 2, plants were dug in December 2020 from the production field and cold‐stored until summer. The varieties used in Field Study 2 were the same as in Field Study 1, with the exception that Corona and Snow White were not used as they were unavailable, and Malling Champion was added. Three batches of six plants of each variety were then grown in troughs in a polytunnel in Cambridge University Botanic Garden with regular irrigation (see Appendix S1 for polytunnel specification and growing medium).

Strawberry flowers are borne on flower stalks: the flower at the terminal of such a stalk is the ‘primary’, has the most carpels and stamens, and usually gives rise to the largest fruits. From the primary branch originate two secondaries, and so on to quaternary and sometimes quinary (McGregor, 1976). Flower position was considered when designing the following experiments.

### 2.2 Measurement of flower size and circularity (Field Study 1)

Flowers from 21 commercial varieties of strawberry were photographed using a SpotCard (Symington & Glover, 2019) to record plant information and to enable automated calculation of flower area, perimeter and circularity. Where possible, photographs were taken from at least 30 flowers from separate plants, but weather and plant development sometimes meant fewer were taken (see Table A2 for *N* values), or multiple flowers from the same plant were used. However, it should be noted that strawberry plants are produced clonally, so genetic variation between plants is minimised. As it was not possible to accurately determine flower age in the field, flowers with fresh‐looking or indehiscent stamens were preferentially chosen: dehiscence occurs within approximately a day of flower opening (McGregor (1976) and personal observation).

After processing with the recognition macro, the detected petal area was manually examined for all photographs and any detection errors manually corrected. Flower circularity was calculated as 4π × area ÷ perimeter^2^.

### 2.3 Measurement of nectar and pollen traits (Field Study 2)

To ensure standardisation of flower age, all flowers were picked and discarded at approximately noon on the first day. On the second day, all open flowers were marked with a patterned label around their stem at approximately noon. On the third day, marked flowers were used for measurement, then all marked flowers picked and discarded, and all unmarked flowers marked with a differently patterned label. This process continued on a rolling basis, ensuring that flowers for measuring were available every morning and that any flowers accidentally left from the previous day were distinguishable. To ensure a plentiful supply of flowers and fair comparison between varieties, only secondary flowers were analysed. Where possible, at least 30 flowers of each variety were analysed over the course of the study, but timing and plant development sometimes meant fewer were taken.

Flowers for nectar and pollen analysis were collected between 0730 and 1200. Flowers were carefully handled to minimise pollen loss, and nectar and pollen measurements were taken within a few minutes of picking. Stamens were counted, then ten were removed and packed into a sterile Eppendorf tube. 150 μL 3:3:2 Tween 80, 0.1% agar and modified Alexanders Stain (Peterson et al., 2010) was added, and the samples stored at −20°C for subsequent pollen counting. Samples were vortexed for 1 min to extract pollen grains from anthers, very briefly centrifuged to collect liquid from the sides of the tube and resuspended with a pipette. Pollen grains were manually counted using a modified Neubauer haemocytometer, with four counts per sample, recording viable (red) and non‐viable (blue) counts simultaneously but separately.

Nectar was collected under a dissecting microscope using 0.5 μL or 1 μL microcapillaries (Drummond, UK). Nectar quantity was measured using a ruler; if enough nectar was collected (in practice above around 0.1 μL), sugar concentration was measured with a low‐volume refractometer (Bellingham and Stanley, UK). Nectar sugar mass was calculated as nectar volume divided by the concentration‐specific density of the solution (density at concentration c (%w/w) = 0.0037291c + 0.0000178c2 + 0.9988603) (Prŷs‐Jones & Corbet, 1987), multiplied by the concentration of the solution.

Temperature was not recorded inside the polytunnel, but the daily maximum and minimum temperatures are manually recorded from the CUBG weather station next to the polytunnel. A weather station nearby (CB3 0FD, 3.3 km away) uploads automated readings every 15 min, and the mean temperatures from that station show very good correlation with the daily maxima recorded at CUBG (data not shown). To calculate the previous day's mean temperature, an average was taken for the 96 readings for each day.

### 2.4 Bumblebee maintenance

*Bombus terrestris audax* bees were supplied by Agralan UK, housed in the nest box in which they were supplied and connected via a gated access tube to a 0.3 × 0.75 × 1.12 m plywood flight arena with a floor painted with Plasti‐Kote fast drying enamel B9 ‘Garden Green’, with a clear UV‐transparent acrylic lid. When not being used for experiments, bees were fed ad libitum with approximately 20% w/w sucrose solution (Tate and Lyle, UK) and supplied with pollen ad libitum in the colony. Bees were marked as previously described (Pattrick et al., 2020).

### 2.5 Artificial flowers for bee experiments

**FIGURE 1:**
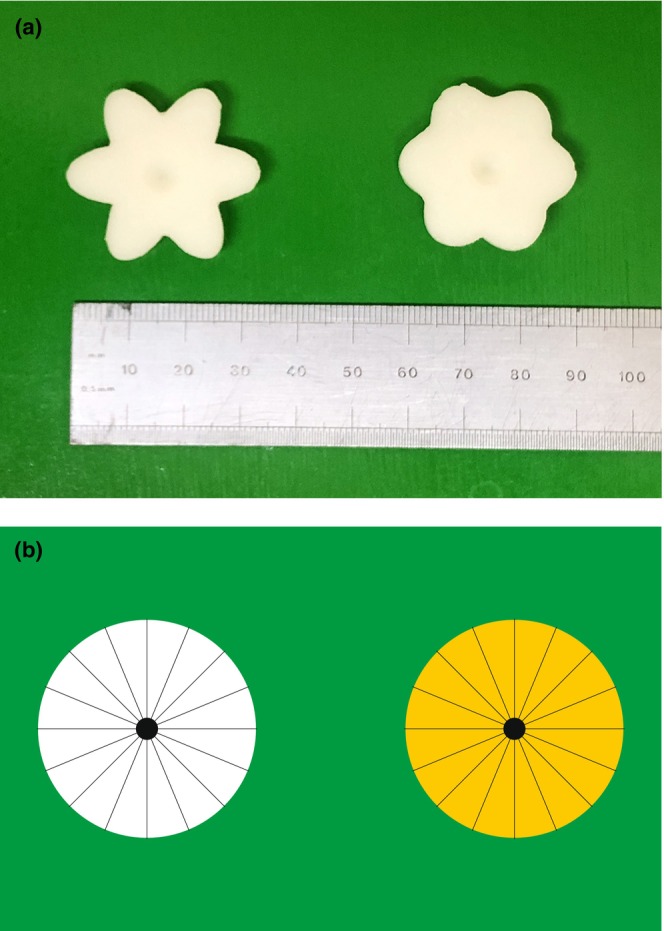
Artificial flowers used for preference experiments. (a) Epoxy‐cast artificial flowers used for differential conditioning experiments. Left: stellated; right: rounder. (b) The card used for testing bumblebee preferences for sugar concentrations at low volumes.

Flower shapes were generated with Adobe Illustrator by overlaying three identical ovals rotated by 0°, 60° and 120° around their central points, giving a shape with six ‘petals’. The resulting composite shape was adjusted so that its area was the same as the median area of all secondary flowers imaged in Field Study 1. The widths of the ovals were adjusted so that the circularity was the same as the 5th or 95th percentile values for circularity of all secondary flowers imaged in Field Study 1. These shapes were laser‐cut from a sheet of plastic, then two cutouts were sandwiched together with superglue, and a piece of 100 grit sandpaper glued to one surface to provide a grippable texture. Casts of these were made by covering them with a premixed polyvinyl siloxane base and catalyst (dental impression mould Elite HD+ light body, normal set, Zhermack) which was allowed to set. Positive artificial flowers (hereafter ‘flowers’) were cast using Gedeo Crystal Resin with 0.66% by mass Titanium White pigment (Cornelissen, London). Once set, a conical indentation (6 mm wide, 2 mm deep) was drilled into the centre of each flower to hold the reward (see Figure 1a).

### 2.6 Innate preference experiments

One approach to improving pollination is to breed flowers which better match pollinator preferences. Bumblebees were therefore tested to determine whether they had an innate preference for either of the extremes of variation seen in the field. Motivated foragers from each of three colonies were selected and trained to associate white objects with a reward over four feeding bouts. One rounder and one stellated flower were placed touching each other immediately in front of the access tube inside the flight arena (first bout), then 10 cm away from the tube and centres 8 cm apart (second bout), then 30 cm away and centres 8 cm apart (third bout), and finally, 60 cm away and centres 8 cm apart (fourth bout), all loaded with 10 μL 40% w/w sucrose solution, replenished when the bee was drinking from the other flower.

For the first innate preference test, two flowers (one of each shape) were placed horizontally on 5 cm tall transparent sample tubes, 20 cm apart and 20 cm from the entrance tube and charged with a 10 μL droplet of a 40% w/w sucrose solution. Trained bees were released, one at a time, and their choices noted. For half of the bees the stellated flower was on the left, and for the other half, it was on the right. The second test was identical in all respects other than that the flowers were placed vertically 90 cm from the entrance tube, with centres 12 cm apart and 7.5 cm from the floor of the arena, supported on two 12 mm wooden dowels attached to a wooden base, painted green (Rust‐Oleum Meadow Green Gloss, Homebase, UK). Flowers were attached with a 1 cm square of Velcro. The third test used the first choice within the differential conditioning experiment, described below.

### 2.7 Differential conditioning experiment

In practice, an improved nectar or pollen reward would have to be coupled with a cue which the pollinator can perceive, to enable the pollinator to distinguish between varieties with different rewards before landing on the flower. Bumblebees were therefore tested to determine whether they could distinguish between the extremes of floral circularity seen in the field. Six pairs of flowers were placed on wooden dowels as above (giving twelve flowers in the flight arena in a 4 × 3 array with centres 12 cm apart between flowers and 25 cm between rows). Flower shape was alternated within rows (i.e. rounder, stellated, rounder, stellated) and the first flower within each row was also alternated (i.e. the first row started with rounder, the second with stellated, the third with rounder). Bumblebees from two colonies (nine from colony 1, 11 from colony 2) were allowed to forage over several feeding bouts, visiting multiple flowers then returning to the colony to deposit the contents of their honey stomach in a holding cell. The alternation pattern of flower shapes was switched for each bout (i.e. if the first flower of the first row was rounder for one bout, for the next it was stellated). One flower shape was loaded with 10 μL 40% w/w sucrose as a reward, the other with 10 μL 0.12% w/w quinine hemisulphate (Sigma, UK), which is distasteful to bees.

Individual bees were allowed to make 100 visits to flowers over multiple feeding bouts. A ‘visit’ was defined as the bee either landing on the flower or flying close enough to it to touch the liquid within it with her antennae. After the test subject had depleted a rewarding flower, it was topped up when she had flown away. Bees did not drink an appreciable quantity of quinine solution. When the bee returned to the colony to deposit the contents of her honey stomach in a holding cell, all flowers were rinsed with water, then 70% ethanol (to remove any scent marks left by the bee), then water again, then patted dry with paper towel. To control for shape preferences, alternate foragers were assigned sugar on stellated flowers and quinine on rounder flowers or vice versa.

### 2.8 Low‐volume nectar concentration experiment

We tested whether previous bumblebee nectar preference studies were relevant at field‐realistic volumes. Several thousand 90 mm × 55 mm disposable cards were printed with two 30‐mm diameter circles, one yellow and one white, on a green background (see Figure 1b). (Preliminary experiments showed that bees can distinguish between these two colours.) The finished cards were laminated with matt plastic to prevent sucrose solutions from soaking into the paper. Circles were charged with 1 μL sucrose solution (either 20% w/w or 35% w/w) using two Eppendorf Multipettes strapped in a positioning jig. These dispense 1 μL aliquots with a manufacturer‐specified inaccuracy (systematic error) of ±1.6% and imprecision (random error) of ±3.0% at 2 μL dispensing volumes (the lowest volume for which these inaccuracies were specified). 15 bees from each of two colonies were trained to associate the coloured circles on the card with a reward (see Appendix S1) before the experiment began. To test the bee, two cards were placed in the flight arena at feeding stations 45 cm apart. The bee was allowed to drink from station 1 and her choice noted. When she flew to station 2, a fresh card was placed at station 1 while her choice at station 2 was noted. This process was repeated until the bee had either taken 25 consecutive drinks from only one of the two circles, or when the bee had taken 500 drinks in total. Fifteen bees from each of two colonies were tested. To control for colour preferences, 15 foragers were assigned the higher concentration on white circles and 15 on the yellow circles. For analysis it was assumed that, once a bee had taken 25 consecutive drinks from the one of the circles, ignoring the other, she would continue to drink solely from that circle.

### 2.9 Statistical analyses

All analyses were performed in R v4.3.0 or v4.3.1 (R Core Team, 2021).

For flower analysis, ANOVAs with Tukey–Kramer HSD post‐hoc analysis was performed using the HSD.test function in the agricolae package v1.3.5.

For bumblebee learning experiments, learning curve data were analysed using binomial logistic regression to fit a fixed effect model to the data, as described in Groen et al. (2016). To encode bee choices in the differential conditioning experiment, 1 and 0 were assigned to a choice from the rewarding flower and the flower with quinine, respectively; in the low‐volume concentration experiment, 1 and 0 were assigned to choices from the high‐ and low‐sugar circles, respectively. χ^2^ tests using Colony as a fixed effect were used to assess whether the fitted models were different to the null model (no learning).

## 3 RESULTS

### 3.1 Flower area varies between varieties

**FIGURE 2:**
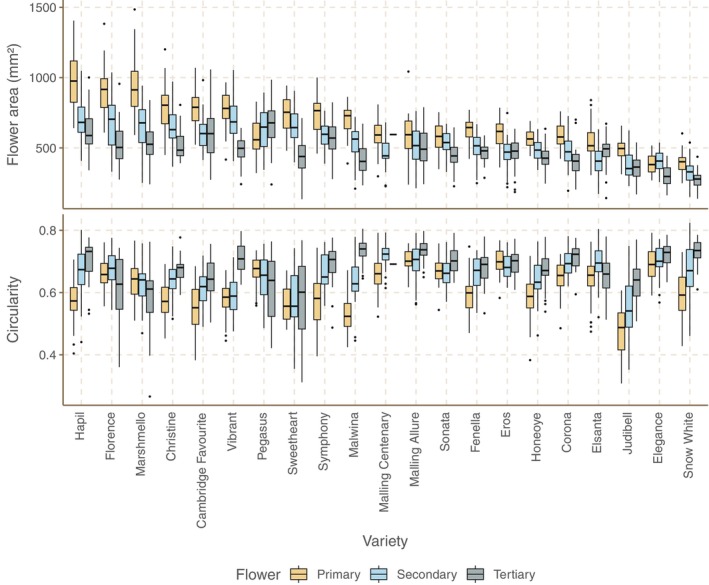
Box plots of flower area and circularity when flattened for primary, secondary and tertiary flowers for each of 21 varieties of strawberry in Field Study 1. Varieties in both plots are sorted by mean primary flower area, allowing for visual comparison of traits across plots. Plot architecture: box: interquartile range (IQR); bar: median; whiskers: to largest/smallest value within 1.5× IQR above/below quartile. See Table A2 for *N* values.

**FIGURE 3:**
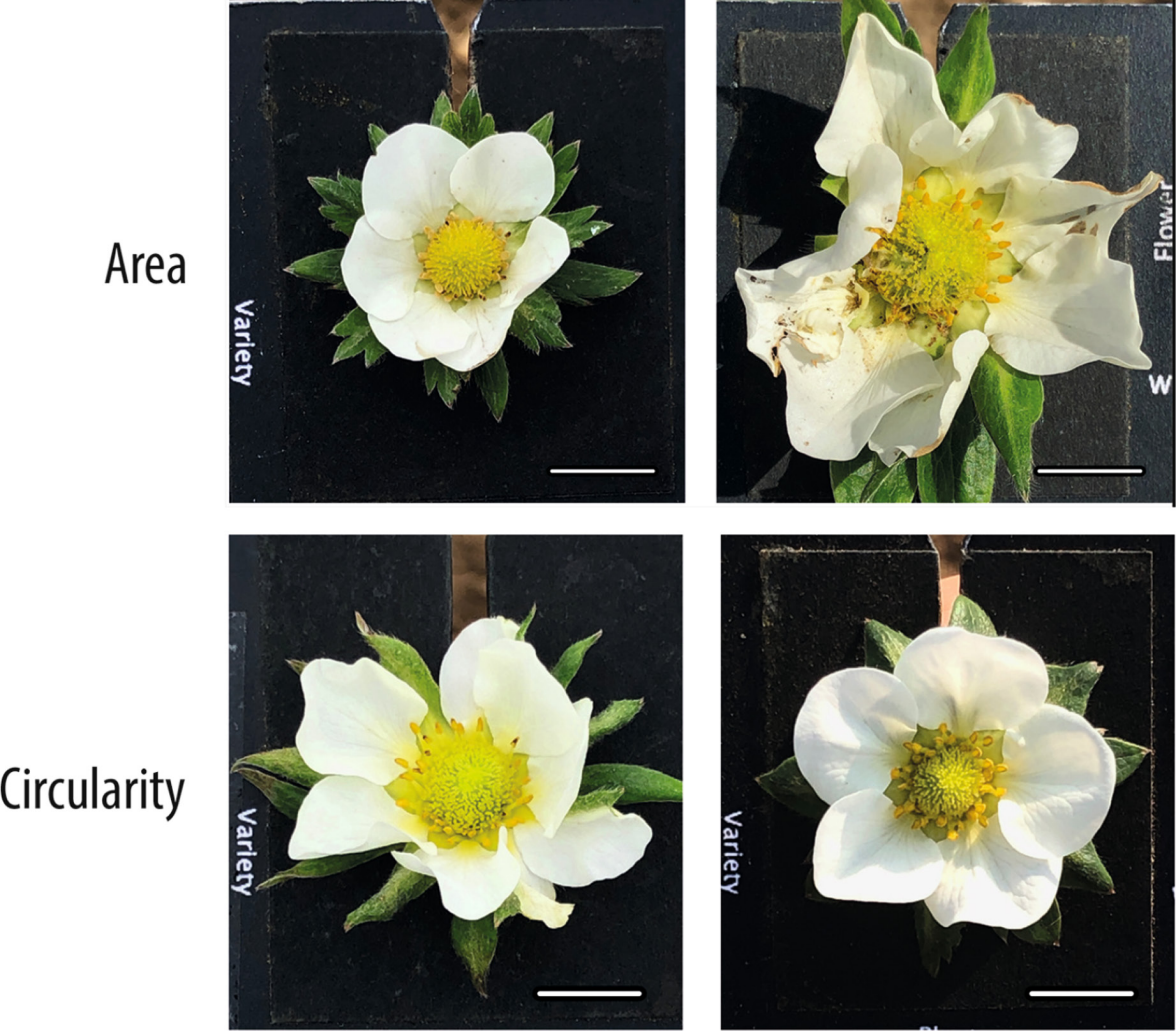
Images of primary flowers displaying median trait values for varieties with the lowest and highest medians for each trait. Flower area medians: 382 mm^2^ (Elegance) to 978 mm^2^ (Hapil); circularity medians: 0.48 (Judibell) to 0.70 (Eros). Scale bars: 1 cm.

Median flower area varied over twofold in each of primary, secondary and tertiary flowers (primary: 382 mm^2^ (Elegance) to 978 mm^2^ (Hapil); secondary: 330 mm^2^ (Snow White) to 704 mm^2^ (Florence); tertiary 277 mm^2^ (Snow White) to 678 mm^2^ (Pegasus)). Secondary flowers were smaller than primary flowers in all varieties except Pegasus and Elegance, and tertiary flowers smaller than secondary flowers in all varieties except Pegasus, Eros, Elsanta and Judibell (see Figures 2 and 3 for representative images).

### 3.2 Floral shape varies between varieties

Floral circularity (calculated as 4π × area ÷ perimeter^2^) varied between varieties (primary: 0.48 (Judibell) to 0.70 (Eros); secondary: 0.54 (Judibell) to 0.72 (Malling Centenary); tertiary: 0.60 (Sweetheart) to 0.74 (Malwina)). There is no significant correlation between flower area and circularity (Pearson's Correlation Coefficient (PCC)/*p*‐value for primary: −.27/.24; secondary: −.21/.35; tertiary: −.31/.16) (see Figures 2 and 3 for representative images).

### 3.3 Strawberry flowers produce tiny quantities of nectar at relatively constant sugar percentages

**FIGURE 4:**
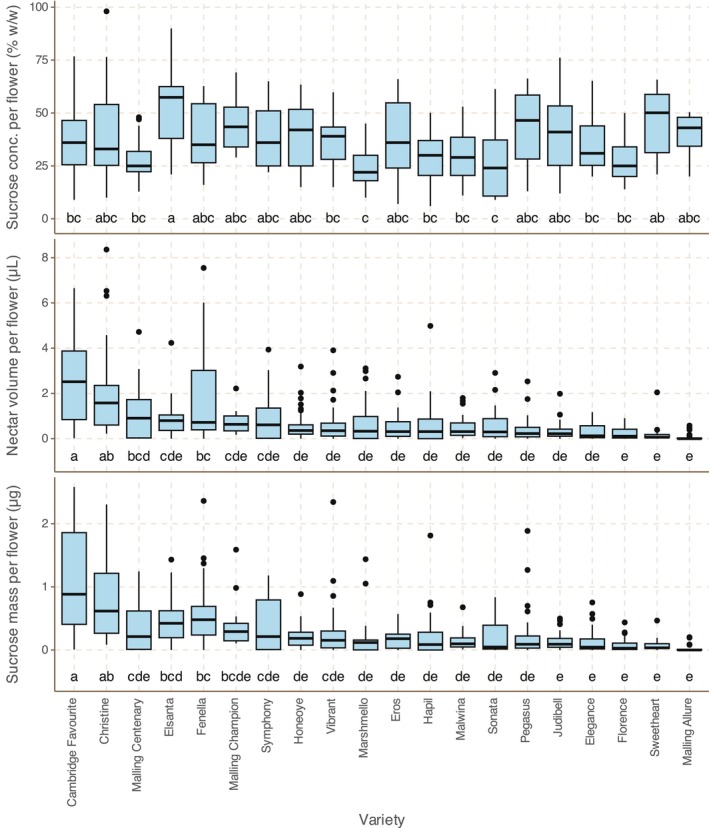
Box plots showing nectar volume, sucrose concentration and sucrose mass by variety for 20 varieties of strawberry. Varieties in all plots are sorted in descending order of median nectar volume per flower, allowing for easy comparison between figures. Plot architecture: box: interquartile range (IQR); bar: median; whiskers: to largest/smallest value within 1.5× IQR above/below quartile. Letters at the base of the plot indicate Tukey–Kramer significance groups (varieties not placed in the same group differ significantly from each other; *p* < .05). See Table A3 for *N* values.

Median nectar production by secondary flowers ranged from 0 μL (Malling Allure) to 2.51 μL (Cambridge Favourite); median sugar concentration ranged from 22% w/w (Marshmello) to 57% (Elsanta). There is more variation between varieties in nectar volume than in nectar sugar concentration, and variation in sugar mass per flower is driven more by nectar volume (PCC between variety medians/*p* value: 0.83/7.2 × 10^−6^) than nectar sugar concentration (PCC between variety medians/*p* value: 0.13/.59). Nectar volume does not correlate with nectar sugar concentration (PCC between variety medians/*p* value: −0.25/.29). There is no correlation between mean secondary flower area and nectar sugar mass or sugar concentration (PCC between variety medians/*p* values: −0.01/.96, −0.24/.31, respectively) (see Figure 4).

### 3.4 Nectar production does not usually depend on temperature

Nectar sugar mass shows a weak positive correlation with temperature across most varieties, which is mostly driven by stronger positive correlations for Cambridge Favourite, Christine, Elsanta and Fenella (PCC/*p* value .56/.00070, .36/.049, .68/1.8 × 10^−6^ and .77/1.2 × 10^−6^, respectively; See Figure A2).

### 3.5 Pollen production and viability varies between varieties

**FIGURE 5:**
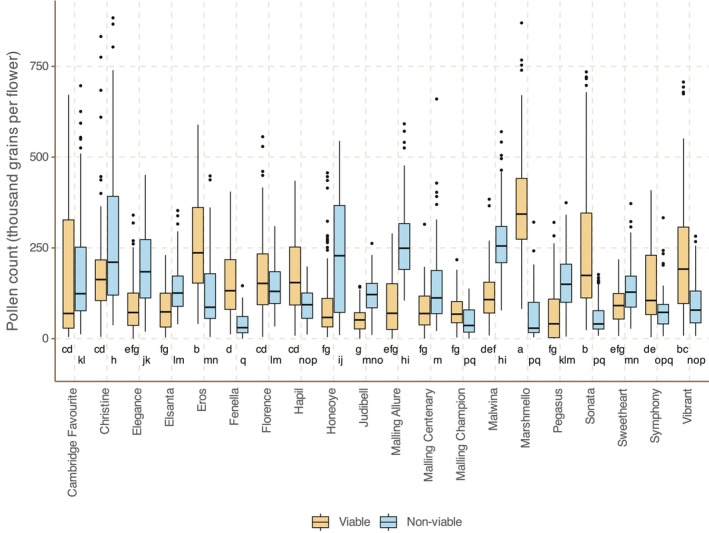
Box plot of number of pollen grains per flower versus variety for Field Study 2, ordered by median number of viable grains. Letters at the base of the plot indicate Tukey–Kramer significance groups (varieties not placed in the same group differ significantly from each other; *p* < .05). Plot architecture: box: interquartile range (IQR); bar: median; whiskers: to largest/smallest value within 1.5× IQR above/below quartile. See Table A3 for *N* values.

The median number of viable pollen grains per secondary flower ranged from 40,875 (Pegasus) to 343,125 (Marshmello); median non‐viable grains ranged from 28,875 (Marshmello) to 255,300 (Malwina). There is weakly negative yet significant correlation between the variety medians for viable and non‐viable grains per flower (PCC/*p* value: −.45/.048; See Figure 5).

### 3.6 Pollen viability does not vary with temperature

Simple linear models of percentage viable pollen versus previous day's temperature show no consistent pattern of variation: correlation is positive for some varieties and negative for others, and there is no temperature above which viability obviously reduces. PCC for observations from each variety all had a modulus of below 0.5, with the exception of observations from Marshmello and Malling Centenary, which showed *positive* correlation between previous day's mean temperature and percentage viability (PCC/*p* value .68/.00070 and .55/.0018, respectively). These data suggest that there is no general correlation, positive or negative, between pollen viability and temperature within the ranges encountered within this study. (See Figure A3).

### 3.7 Field‐realistic variation in floral circularity is at the limit of bumblebee detection

**FIGURE 6:**
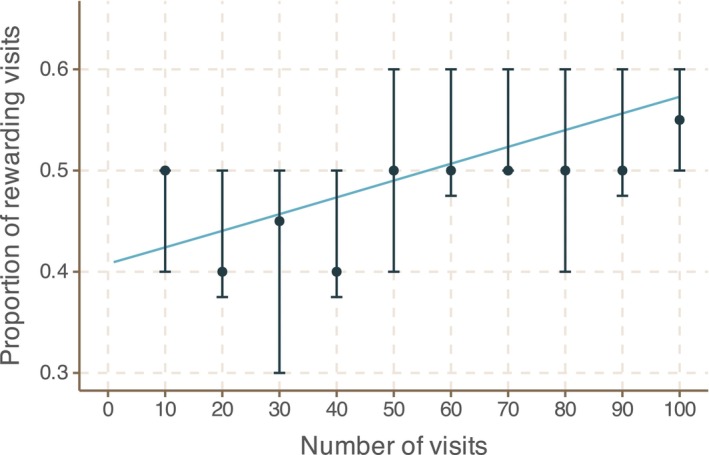
The ability of *B. terrestris* to distinguish stellated from round flowers. Bees were tested for the ability to associate the rewards with shape stimuli. Data are shown pooled over all bees (*n* = 20) into successive groups of ten choices, with points showing means and error bars showing the 25% and 75% quantiles for the proportion of correct choices. The blue curve shows the fitted binomial logistic model. The χ^2^ statistic and *p*‐value for the likelihood ratio test assessing whether or not bees are able to learn are 18.34 and 1.85 × 10^−5^, respectively.

When stimulus differences are obvious to bees, they can learn to around 90% accuracy within 100 visits (e.g. Groen et al. (2016), personal observation and see also the low‐volume nectar concentration experiment later in this manuscript). Here, individual bees were able to learn over 100 choices to distinguish between rounder and stellated flowers, though this learning was very slow (see Figure 6; logistic regression χ^2^ statistic 18.34 and *p*‐value 1.85 × 10^−5^). This suggests the variation in shape is at the limit of that which can be detected by bumblebees.

### 3.8 Bumblebees do not conclusively display an innate preference for flower shape

In the first test of innate preference, 22 of 30 bees chose to forage from the stellated flower, while eight of the 30 bees chose the rounder flower—a clear deviation from random choice (*n* = 30, *p* = .0081, binomial test). In the second test, 13 of 30 bees chose to forage from the stellated flower, while 17 chose the rounder flower, showing no deviation from random choice (*n* = 30, *p* = .57, binomial test). In the third test, 10 of 30 bees chose to forage from the stellated flower, just over the significance threshold (*n* = 30, *p* = .049, binomial test). Combining all three experiments, 45 of 90 bees—exactly half—chose to forage from the stellated flower. Taken together, these results indicate that bumblebees do not have a preference for flower shape within the limits of observed variation.

### 3.9 Bumblebees can distinguish between 20% and 35% sugar solutions at field‐realistic volumes

**FIGURE 7:**
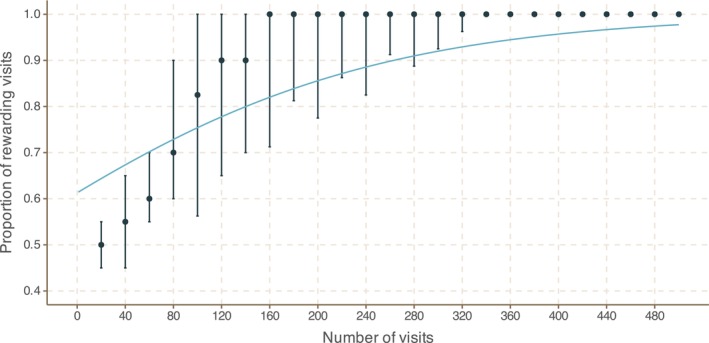
The preference of *B. terrestris* for 35% over 20% w/w sugar solution at 1 μL quantities. Choices were recorded as the bees learned to associate the rewards with colour stimuli. Data are shown pooled over all bees (*n* = 30) into successive groups of 20 choices, with points showing means and error bars showing the 25% and 75% quantiles for the proportion of correct choices. The blue curve shows the fitted binomial logistic model. For analysis it was assumed that, once a bee had taken 25 consecutive drinks from the one of the circles, ignoring the other, she would continue to drink solely from that circle. The χ^2^ statistic and *p*‐value for the likelihood ratio test assessing whether or not bees are able to learn are 1364 and 1.08 × 10^−298^, respectively.

All bees were able to learn which colour circle presented the higher‐sugar reward (logistic regression χ^2^ statistic 1364 and *p*‐value 1.08 × 10^−298^ and see Figure 7). All 15 bees from Colony 1 and nine of 15 bees from Colony 2 eventually foraged solely from the more‐rewarding circle for 25 consecutive visits, while six bees from Colony 2 often foraged from just one circle on each visit to a card, but continued to forage periodically from both circles on the same card.

Bees' learning speeds varied: of the 24 bees which chose to forage solely from one circle, the fastest completed the experiment in 42 drinks, while the slowest took 370 drinks. The slower‐to‐learn bees displayed a general preference for the more‐rewarding circles within 100–150 drinks, but continued to drink from the less‐rewarding circles from time to time.

## 4 DISCUSSION

Breeding plants to select for a particular trait is predicated on the existence of genetically determined variation in that trait. This study has identified significant variation between genetically distinct varieties of strawberry in flower area, flower circularity, nectar volume, pollen production and pollen viability and shown that there is little correlation between trait measurements. This suggests that breeding for improvement in these traits would be feasible and that they could be bred for independently. Given that variation in floral shape variation was at the limit of that which can be detected by bumblebees, it would be appropriate to focus any breeding efforts on improving floral reward.

### 4.1 Flower size and shape

To the best of our knowledge, this study is the first to measure strawberry flower size to any degree of accuracy. Primary flower area was found to range from 382 to 978 mm^2^, which corresponds to 22 to 34 mm in diameter, assuming flowers are a perfect circle. This agrees well with a 1976 description, quoting sizes from 25 to 40 mm (McGregor, 1976). Secondary flowers in this study were generally smaller than primary flowers, which also corresponds with previous description (McGregor, 1976).

It is obvious that it will be easier for an insect to see a larger flower than a smaller one, assuming flower colour and distance from the insect are identical. Breeding for larger flowers would, therefore, be a simple way to increase floral visibility, but this may have unwanted effects on consumer‐relevant traits, particularly fruit size. As well as being more visible, larger flowers are also preferred by bees, as demonstrated in, for example, *Polemonium* (Galen & Newport, 1987) and *Raphanus* (Stanton & Preston, 1988). This is possibly because they are easier to see, but perhaps because larger flowers may produce more by way of a nectar reward, as demonstrated in the same two species by Cresswell and Galen (1991) and Stanton and Preston (1988) and by Eisen et al. (2023) in *Arabis alpina*. However, the current study found no correlation between median secondary flower area and median nectar production across varieties. It would be interesting to measure nectar production using variously sized flowers from a single variety of strawberry, to better probe the effect of flower size on nectar production.

Selecting for flowers which are a better match for pollinator preferences or which are easier for them to see will improve pollinator visitation rates. After flower size, flower shape is the most obvious visual trait which could have relevance to pollinators, and it has previously been shown that pollinators prefer strawberry flowers with longer petals (Ashman, 2000). The current study found that bumblebees did not have a preference for flowers with circularity at the extremes of that found in the field (but with identical areas), but that they could just discern the difference between them in conditioning tests. This supports previous work determining that the resolution of bee vision is up to around 0.6° (Hempel de Ibarra et al., 2015; Rigosi et al., 2017), which corresponds to objects approximately 1 mm wide at 10 cm distance. However, bees in this study learned very slowly, implying that the difference in floral shape is at the limit of that which can be detected by bees: if the stimulus is easily detectable by bees, they learn much faster (e.g. iridescence in *Hibiscus trionum*, learnt to near‐total accuracy within around 75 choices (Moyroud et al., 2017), or floral temperature patterning, learnt to around 80% correct over 60 choices (Harrap et al., 2017)).

### 4.2 Nectar

Nectar production fell broadly within the limits of an earlier study in strawberry (0.02–0.73 μL flower^−1^ day^−1^ (Abrol, 1992)); some varieties and flowers in this study produced more than the quantities reported by Abrol. These quantities are extremely small. A honeybee colony requires around 335 kg nectar (30% w/w sucrose) per year simply to survive, without producing any surplus honey (Rodney & Purdy, 2020). If a strawberry flower produces 1 μL nectar over its lifetime—at the upper end of production observed in this study—it would take 297 million strawberry flowers to provide for the colony, meaning the entire UK strawberry crop of 2021 (FAO, 2022) would have provided the nectar requirements of 36 honeybee colonies. A strawberry flower with a daily nectar production of 0.5 μL nectar at 30% w/w sugar equates to 0.17 μg sugar per day; in a study measuring nectar in 175 species (Baude et al., 2016), 91% of species surveyed produced more than this. By contrast, flowers of bramble (*Rubus fruticosus*), a common British hedgerow plant, produced 1900 μg sugar per day—3400 times more than strawberry flowers.

Nectar sugar concentration in this study ranged from medians of 22% to 50%, in contrast to 30–42% in the previous study and within the limits of (though generally lower than the values identified in) a comparable study in field beans (Bailes et al., 2018). Pattrick et al. (2020) showed that there is only a small, non‐significant difference between the volumetric rates at which *B. terrestris* drink 35% w/w sucrose solution and 50% w/w sucrose solution; Harder (1986) conducted a study with 22 bees of nine species, showing that drinking rate was constant up to 40% w/w sucrose and only 20% slower at 50% sucrose: increasing sugar concentration from 10% to 40% and leaving nectar volume unchanged would have no effect on the time spent visiting the flower. Increasing the volume of nectar produced by individual flowers would reduce the time spent flying between flowers by insects on foraging trips, but this would also result in a reduction in the number of flowers visited per foraging trip to collect a given volume of nectar. This would conflict with the aim of helping insects visit more flowers more quickly—there is clearly a balance to be struck.

Bees discriminated between nectar of 20% and 35% w/w sucrose at microlitre volumes, ignoring the 20% sucrose either entirely or almost entirely once they had learned to associate concentration with a visual cue. This confirms that previous observations that bees prefer higher‐sugar nectar to lower‐sugar nectar (e.g. Bailes et al. (2018)) are valid at field‐realistic volumes, and agrees with the finding of Cnaani et al. (2006), where bees preferentially chose lower‐volume, higher‐concentration rewards. It only takes a second or two for a bee to drink 1 μL nectar, so it is interesting that they will choose to essentially double their foraging time by only drinking one of the two rewards on offer, rather than returning to the colony with a load of nectar which is slightly less rich in energy.

### 4.3 Pollen

Pollen production in this study ranged from 104,000 to 373,000 grains per flower. This fits well with the study by Ariza et al. (2015), which measured pollen production in the strawberry variety Camarosa, which produced 249,000 grains per flower. The most comprehensive study of pollen production in different UK species is by Hicks et al. (2016), but they quantified pollen in terms of volume rather than count: larger grains will contain more protein than smaller grains. Viable strawberry pollen grains can be approximated as spheres with diameter 0.025 mm (Ariza et al. (2015) and microscopy images from this study (data not shown)); using the same methodology as Hicks et al. (2016) to calculate pollen volume per strawberry flower, assuming gapless packing of pollen grains, gave a volume of 2.8 μL pollen per flower for Marshmello, the highest‐producing variety, larger than most weeds analysed by Hicks et al. (2016) and comparable with dandelion (*Taraxacum* agg.) and rosebay willowherb (*Chamerion angustifolium*).

Pollen viability also varied, with percentage viability ranging from 20% (Honeoye) to 92% (Marshmello). Viability in Camarosa has previously been reported as 85% (Ariza et al., 2015). Strawberry pollen viability is known to be affected by temperature in some cultivars (Ledesma & Sugiyama, 2005; Leech et al., 2000), but this study found no general correlation between temperature and viability as measured by staining. In those studies, plants were held at high temperatures for several days, and it is possible that in this study the lower night temperatures were low enough to permit repair of damage sustained during the higher daytime temperatures or that some other variable mitigated loss of viability. (This study did not test germination as the focus is on bee‐relevant floral traits.)

This study did not test bumblebee preferences for pollen quantity or viability, and there are relatively few reports of such tests in comparison to the wider body of knowledge of bumblebee responses to nectar‐based variables. Robertson et al. (1999) used genotypes of *Mimulus guttatus* which differed in pollen production to show that bumblebees preferentially visited plants that had high numbers of viable pollen but an otherwise low total pollen production.

There is also a general lack of studies addressing the question of whether bees display preferences for flowers displaying high or low quantities of pollen. This is perhaps because constructing artificial flowers to dispense precisely controlled quantities of pollen is difficult in comparison to dispensing ‘nectar’ (see Russell and Papaj (2016) for some discussion). In lab experiments, Konzmann and Lunau (2014) found bumblebees would collect whatever pollen was available regardless of quality, but in that case quantity was held constant, bulking out *Pinus* pollen with powdered cellulose or glass. By contrast, fieldwork suggests bumblebees focus on pollen quality rather than quantity (Leonhardt & Blüthgen, 2012) or protein:lipid ratio (Vaudo et al., 2016).

There is clearly much to learn about bumblebee behaviour in relation to pollen. However, regardless of bee preferences, it is obvious that having a larger number of pollen grains available for collection will increase the amount of food available to pollinators. Additionally, having a larger percentage of viable pollen is likely to improve autonomous and animal‐mediated self‐ and cross‐pollination.

One potential way of increasing pollen production within a flower could be to increase the number of stamens on that flower. Interestingly, stamen number in strawberry is heavily linked to ploidy. A study many years ago using assorted diploid, tetraploid, hexaploid, octaploid, decaploid and dodecaploid *Fragaria* species and the related *Potentilla indica* showed that plants in the 2×, 4×, 6× and 12× ploidy groups presented on average around 20 stamens, while those in the 8× ploidy group presented around 35, and those in the 10× ploidy group presented around 30 (Haskell & Williams, 1954). In that study, the authors also mention work in diploid and tetraploid cherries, showing that stamen number and variability has a genetic basis. Increasing the ploidy of crop plants is hardly a practical strategy for improving pollinator‐relevant traits, given the numerous effects of polyploidy on the rest of the plant's architecture, vegetative traits and metabolome (Trojak‐Goluch et al., 2021). However, it may be that within these species in the Rosaceae, selection for consumer‐relevant traits has inadvertently given rise to flowers which are better for pollinators, at least in terms of pollen.

### 4.4 Pollinator assemblages

This study used bumblebees as a model organism due to their tractability. In the wild, strawberries are pollinated by a range of insects: in various studies, honeybees, syrphids (hoverflies) and halictids (sweat bees) were observed to visit in varying proportions in Québec (de Oliveira et al., 1991), Utah (Nye & Anderson, 1974) and India (Abrol et al., 2017). Chagnon et al. (1993) found that honeybees and small bees of other species played a complementary role in pollination: honeybees pollinated stigmata at the apex of the gynoecium, while the smaller bees pollinated mainly the basal stigmata.

When considering whether our data can be extrapolated to this wider pool of insects, it seems reasonable to expect that changes in relatively high‐level floral traits (e.g. reward or floral size) are more likely to produce similar effects across a range of insects than would be produced with fine‐grained changes (e.g. subtle variation in floral shape). While insects of different species drink at different speeds (Kim & Bush, 2012), it is also reasonable to expect that increasing nectar quantity or concentration would generally benefit insects due to the increased energy provided in that nectar. Similarly, improving the nutritional value or quantity of pollen would likely benefit all pollinators.

## 5 CONCLUSIONS

This study has identified significant differences in numerous floral traits in strawberry. Because the bumblebees were only just able to detect field‐realistic variation in floral shape, and because nectar sugar concentration has limited variation, breeding for these traits is likely to have limited effect. However, pollen production has the potential to be of relevance to pollinating insects: it consistently varies between varieties, so it is clear that there is a genetic basis to the variation. Strawberries could be bred for increased pollen production and pollen viability, which would likely improve pollination. However, we lack knowledge of whether this increased production and viability would be beneficial to pollinating insects.

Additionally, there is the question of precisely how pollen production is genetically determined: selection for increased pollen production or pollen viability could have pleiotropic effects. This could be addressed with breeding and quantification experiments, assisted by quantitative trait locus (QTL) analysis, which in strawberry could use existing single‐nucleotide polymorphism (SNP) arrays: the iStraw90k and iStraw35k offer 95,062 and 34,260 markers, respectively (ThermoFisher, 2022). A previous study has already addressed SNPs associated with male sterility (Wada et al., 2020), but this treated this sterility as a binary variable. It would be interesting to sequence the varieties tested in this study and analyse QTLs associated with the variability in pollen count and viability.

Thinking ahead to field application, it is important to note that consideration should be given to insect visitation rates when growing several varieties in adjacent rows in the same field. Situating a variety with flowers preferred by bees next to another variety with less‐preferable flowers may drain pollinators from the less‐favoured variety.

## AUTHOR CONTRIBUTIONS

**Hamish A. Symington:** Conceptualization (lead); formal analysis (lead); investigation (lead); methodology (lead); writing – original draft (lead); writing – review and editing (lead). **Beverley J. Glover:** Conceptualization (supporting); methodology (supporting); project administration (lead); supervision (lead); writing – review and editing (supporting).

## FUNDING INFORMATION

HS was funded by the BBSRC Doctoral Training Partnership at the University of Cambridge, grant number BB/M011194/1.

## CONFLICT OF INTEREST STATEMENT

The authors declare that the research was conducted in the absence of any commercial or financial relationships that could be construed as a potential conflict of interest.

## Supporting information


Appendix S1

## Data Availability

Data files associated with this publication are available from https://doi.org/10.17863/CAM.101779.
