# Correction to “Strawberry Varieties Differ in Pollinator‐Relevant Floral Traits”

**DOI:** 10.1002/ece3.70842

**Published:** 2025-01-08

Symington, H. A., and B. J. Glover. 2024. “Strawberry Varieties Differ in Pollinator‐Relevant Floral Traits.” *Ecology and Evolution* 14: e10914. https://doi.org/10.1002/ece3.10914.

In the Data Availability Statement, the link to the data files associated with this publication was incorrect. The data files can be found at https://doi.org/10.17863/CAM.101779. The online version of this article has been corrected accordingly.

We apologize for this error.

