# Supplementary material for: Strawberry varieties differ in pollinator‐relevant floral traits
Source: Ecol Evol. 2024 Feb 5;14(2):e10914. doi: 10.1002/ece3.10914 (PMC10844710; doi:10.1002/ece3.10914)
Supplement: Supplementary file 1 — Appendix S1 [file ECE3-14-e10914-s001.pdf]

Appendix

Field study 1: field specifications

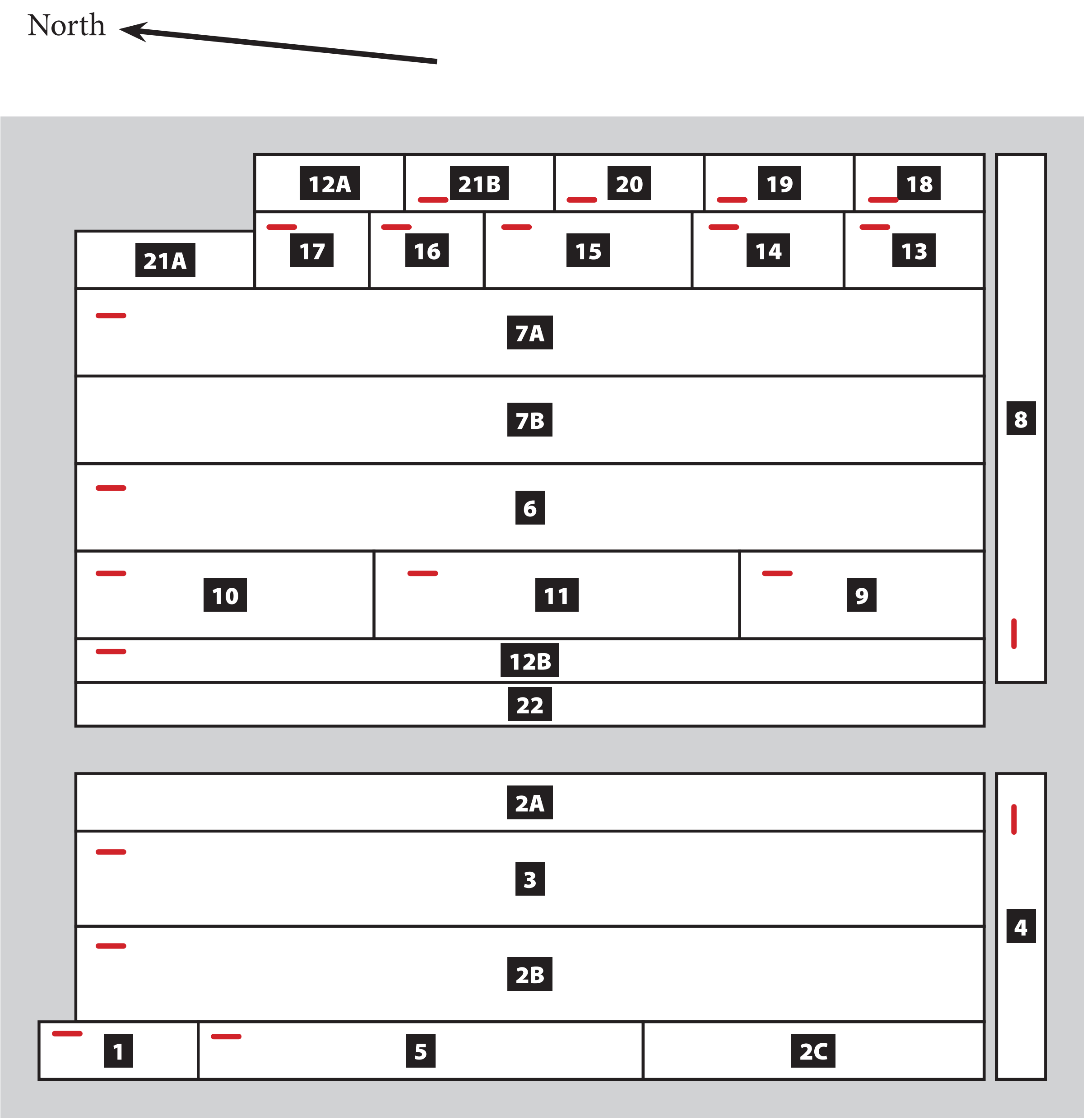

Figure A1: The layout of the field in East Winch used in Field Study 1, approximately to scale. The distance from block 1 to block 12a (*i.e.* the width of the field) is approximately 200 m; the larger blocks (*e.g.* 7A, 7B) are approximately 22 m wide and 200 m long. Numbers indicate the variety of strawberry grown in each block; letters indicate when the same variety was grown in multiple blocks. Within each block, rows run left to right (*i.e.* approximately north to south). See Table A1 for the key to block planting.

Number	Block	Variety	Rows in block	Plants in block
1	–	Snow White	12	1,000
2	A	Malwina	10	5,500
2	B	Malwina	24	13,300
2	C	Malwina	12	2,500
3	–	Malling Allure	24	13,300
4	–	Korona	12	2,000
5	–	Eros	12	3,000
6	–	Vibrant	24	13,400
7	A	Malling Centenary	24	13,400
7	B	Malling Centenary	24	13,400
8	–	Elsanta	12	5,000
9	–	Fenella	24	3,600
10	–	Florence	24	4,500
11	–	Elegance	24	5,000
12	A	Christine	12	1,000
12	B	Christine	6	3,000
13	–	Symphony	24	2,100
14	–	Sonata	24	2,000
15	–	Cambridge Favourite	24	2,750
16	–	Honeoye	24	1,500
17	–	Sweetheart	24	1,500
18	–	Pegasus	12	1,000
19	–	Hapil	12	1,000
20	–	Judibell	12	1,000
21	A	Marshmello	12	1,000
21	B	Marshmello	12	650
22	–	Malling Opal	6	3,500

Table A1: Key to the layout of the field in East Winch used in Field Study 1.

Field Study 1 spray applications

Field Study 1 was conducted on a working farm, and fertilizer, herbicide and fungicide sprays are routinely used. Applications during the growing period for these plants were:

- 2 January 2019: Isoxaben (500 g L^{-1} in Flexidor), broad-spectrum herbicide (dose: 0.4 L ha^{-1} in 200 L ha^{-1} water)
- 13 March 2019: Phenmedipham (160 g L^{-1} in Betasana), broad-leaved herbicide (dose: 2 L ha^{-1} in 200 L ha^{-1} water)
- 30 March 2019: Phenmedipham (160 g L^{-1} in Betasana), broad-leaved herbicide (dose: 2 L ha^{-1} in 200 L ha^{-1} water)

Variety	Primary	Secondary	Tertiary
Cambridge Favourite	31	31	30
Christine	31	32	15
Corona	30	32	21
Elegance	30	32	30
Elsanta	56	51	30
Eros	33	32	33
Fenella	30	32	31
Florence	31	31	31
Hapil	32	27	31
Honeoye	30	30	30
Judibell	29	32	32
Malling Allure	33	30	30
Malling Centenary	30	34	1
Malwina	31	31	32
Marshmello	29	38	29
Pegasus	31	33	31
Snow White	12	34	40
Sonata	31	32	30
Sweetheart	31	32	31
Symphony	31	29	31
Vibrant	33	31	30
**Totals**	**655**	**686**	**599**

Table A2: N values for all varieties and positions of flowers measured in Field Study 1.

Field study 2: growth specification

Three batches of six plants of each variety were grown in rows of plastic troughs (1 m × 18 cm × 10 cm), supported on an identical, upturned trough, with 30 cm between rows. Four plants were grown in each trough in a substrate comprising 75% coir, 15% sterilised loam and 10% angular grit, supplemented with 4.5 kg Osmocote 7/9 months 15–9–11 NPK + 2MgO + TE, 1 kg Magnesian limestone and 100 mL H2Gro wetting agent in 100 L water per tonne ('CUBG Mix 2', Hewitt Sportsturf, Leicester). The troughs were housed in a 12 ft × 35 ft polytunnel (First Tunnels, Lancashire, UK) covered with Super Therm polythene, with side ventilation constructed from 1 mm insect-proof mesh on all sides. Plants were supplied with water from a borehole via soaker hose for one minute every two hours to keep the soil moist but not wet.

N values for nectar and pollen measurements in Field Study 2

Variety	Flowers sampled for nectar volume	Flowers for which nectar sugar concentration was determined	Flowers sampled for pollen count
Cambridge Favourite	33	30	30
Christine	30	28	29
Elegance	32	18	27
Elsanta	38	32	24
Eros	31	23	29
Fenella	29	26	29
Florence	32	17	32
Hapil	31	19	30
Honeoye	40	35	32
Judibell	35	25	26
Malling Allure	32	7	27
Malling Centenary	32	22	30
Malling Champion	12	12	12
Malwina	31	23	13
Marshmello	23	13	21
Pegasus	42	30	26
Sonata	31	24	30
Sweetheart	31	12	32
Symphony	31	21	30
Vibrant	30	23	30

Table A3: N values for flowers used for nectar and pollen samples in Field Study 2. In practice, it was impossible to obtain refractometer readings when nectar volumes were below around 0.15 μL; for flowers which did not produce enough nectar to determine the sugar concentration, the mean sugar concentration for all measurements from flowers from that variety was used.

Bee training

Low-volume experiment

Bees were trained to associate the coloured circles on the card with a reward over five training bouts. A card was placed in the flight arena, immediately outside the entrance tube. For the first two bouts, several droplets of approximately 10 μL were placed on both circles; for the third to fifth bout, similar sized droplets were placed in the centre of both circles; empty circles were replenished while the bee was drinking from the other circle.

Differentiation experiment

Motivated foragers were selected and trained to associate white objects with a reward over four feeding bouts (with one rounder and one stellated flower placed touching each other immediately in front of the access tube inside the flight arena, then 10 cm away from the tube and centres 8 cm apart, then 30 cm away and centres 8 cm apart, and finally 60 cm away and centres 8 cm apart, all loaded with 10 μL 40% w/w sucrose solution, replenished when the bee was drinking from the other flower).

Nectar sucrose mass vs temperature

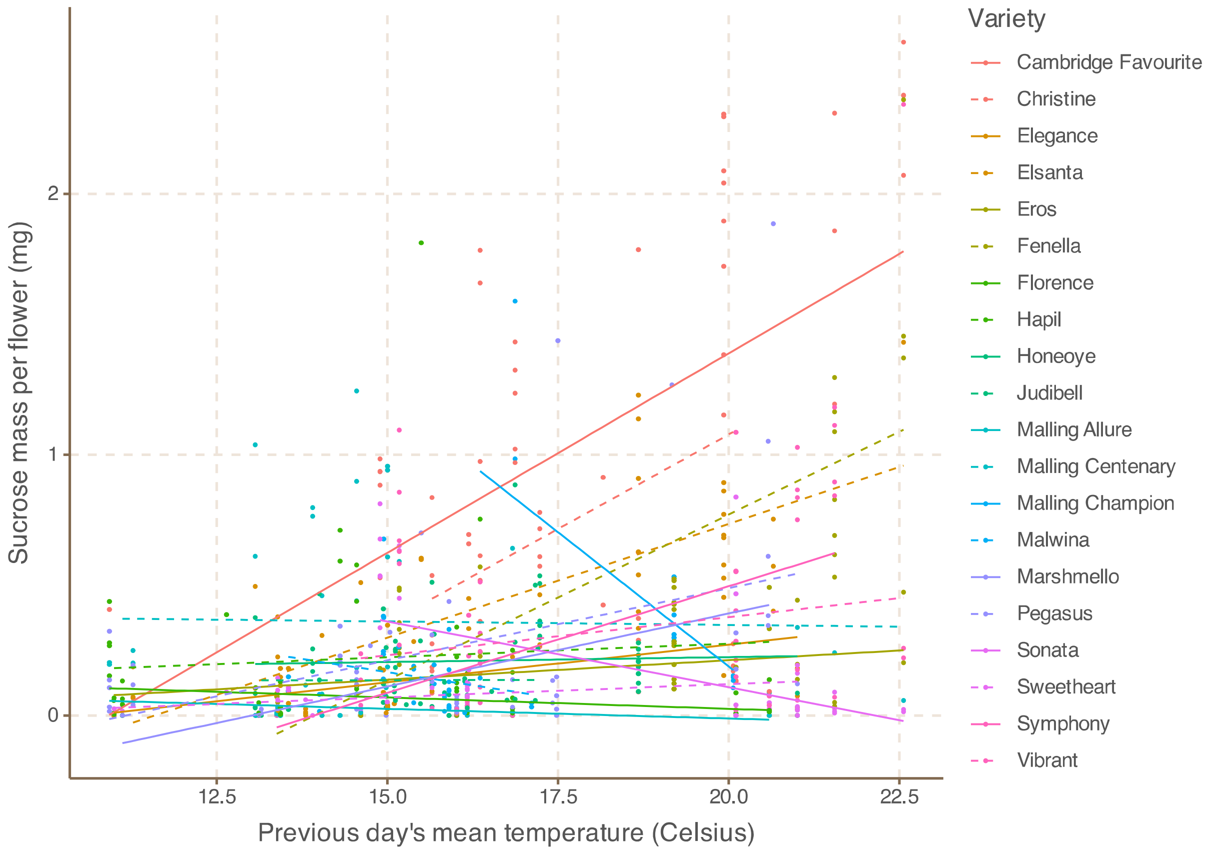

Figure A2: Scatter plot of sucrose mass per flower *vs* previous day's mean temperature for Field Study 2. Cambridge Favourite, Elsanta and Fenella are the only varieties for which there are strong correlations between sucrose mass per flower and previous day's mean temperature *and* for which there is a reasonably good fit for the linear model. See Table A3 for N values.

538 # Pollen viability vs temperature

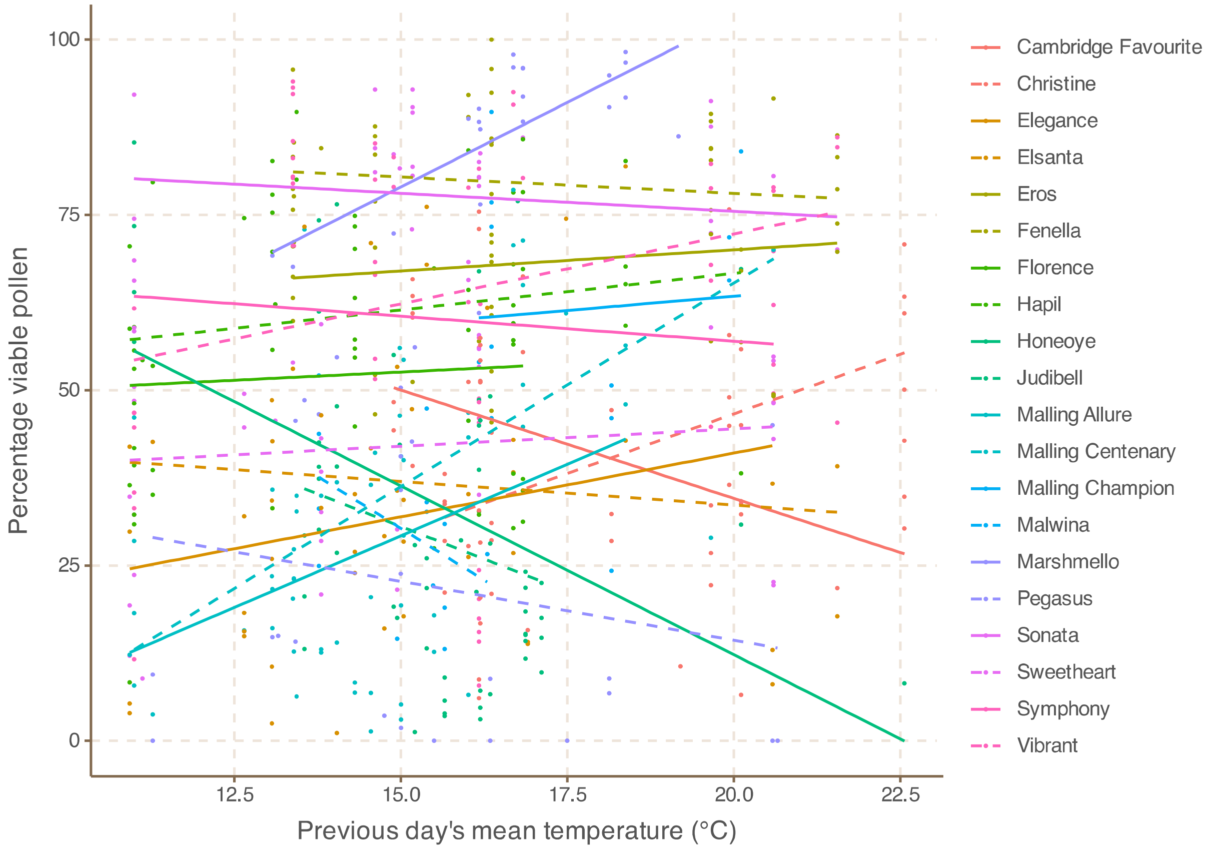

Figure A3: Scatter plot of percentage viable pollen (one data point for the mean of the four counts per flower per variety per day) *vs* previous day's mean temperature for Field Study 2. Marshmello is the only variety for which there is a strong correlation between percentage viable pollen and previous day's mean temperature *and* for which there is a reasonably good fit for the linear model (visible as the mauve line with a positive slope at the top of the plot). See Table A3 for N values.
